# Febuxostat attenuates ER stress mediated kidney injury in a rat model of hyperuricemic nephropathy

**DOI:** 10.18632/oncotarget.22784

**Published:** 2017-11-30

**Authors:** Li He, Ying Fan, Wenzhen Xiao, Teng Chen, Jiejun Wen, Yang Dong, Yiyun Wang, Shiqi Li, Rui Xue, Liyang Zheng, John Cijiang He, Niansong Wang

**Affiliations:** ^1^ Department of Nephrology, Shanghai Jiao Tong University Affiliated Sixth People’s Hospital, Shanghai, China; ^2^ Department of Medicine, Division of Nephrology, Icahn School of Medicine at Mount Sinai, New York, NY, USA

**Keywords:** hyperuricemic nephropathy, hyperuricemia, ER stress, renal tubular cells, Febuxostat

## Abstract

Hyperuricemia contributes to kidney tubular injury and kidney fibrosis. However, the underlying mechanism remains unclear. Here we examined the role of RTN1A, a novel endoplasmic reticulum (ER)-associated protein and ER stress in hyperuricemic nephropathy. We first found the expression of RTN1A and ER stress markers was significantly increased in kidney biopsies of hyperuricemia patients with kidney injury. In a rat model of hyperuricemic nephropathy (HN) established by oral administration of a mixture of adenine and potassium oxonate, increased expression of RTN1A and ER stress was shown in tubular and interstitial compartment of rat kidneys. Treatment of Febuxostat, a new selective inhibitor of xanthine oxidase (XO), not only attenuated renal tubular injury and tubulointerstitial fibrosis, but also reduced uric acid crystals deposition in HN rat kidneys. *In vitro*, Febuxostat also reduced ER stress and apoptosis in uric acid treated tubular epithelial cells. Our data suggest that RTN1A and ER stress mediate tubular cell injury and kidney fibrosis in HN. Urate-lowering therapy (ULT) with Febuxostat attenuates uric-acid induced ER stress in renal tubular cells and the progression of HN.

## INTRODUCTION

Increasing evidence indicates that hyperuricemia is an independent risk factor for the onset and progression of chronic kidney disease (CKD)[1]. It has been well recognized that hyperuricemia impairs the kidney through the obstruction of renal tubules with urate crystal deposition, resulting in the renal tubular cell injury and subsequent interstitial fibrosis [2, 3]. Studies showed that endothelial dysfunction, oxidative stress, inflammation and the activation of renin-angiotensin system may be involved in the hyperuricemia induced kidney injury [3–7]. However, the exact mechanism of hyperuricemic nephropathy (HN) and how hyperuricemia contributes to the progression of HN remains largely unknown.

Febuxostat, an oral inhibitor of xanthine oxidase (XO), has been shown to be effective at reducing serum uric acid (SUA) levels [8, 9]. In 2009 Febuxostat was approved by the U.S. Food and Drug Administration, opening a new era for the treatment of gout [10]. Since then, quite a few randomized multi-central trials have confirmed a potent role of Febuxostat as urate-lowering therapy (ULT) in hyperuricemia induced gout [11, 12]. Recent studies suggested that lowering SUA levels by Febuxostat might also contribute to cardiovascular and renal benefits [13]. However, the mechanism of this protective effect is largely unknown.

A variety of damages such as hypoxia, ischemia and hyperglycemia may disturb Endoplasmic reticulum (ER) homeostasis, which can induce ER stress and the subsequent unfolded protein response (UPR)[14, 15]. ER stress at early stage has an adaptive role in restoring the homeostasis and protect cells against injury, however, excessively prolonged ER stress will finally cause cell death by triggering pro-apoptotic signaling pathways [16]. ER stress has been shown to contribute to the development and progression of chronic kidney disease (CKD)[17, 18]. Increased expression of ER stress marker GRP78 was observed in murine models of minimal-change nephrotic syndrome and passive Heymann nephritis [19, 20]. Knockdown of CHOP expression *in vitro* decreased HSA-induced apoptosis of proximal tubular cells [16]. In our previous studies, we identified a novel ER-associated gene, reticulon-1A (RTN1A), which is associated with the progression of kidney diseases such as diabetic nephropathy and HIV-associated nephropathy (HIVAN)[16, 21]. Recently, we reported that RTN1A and ER stress are involved in the development of acute kidney injury (AKI) and progression from AKI to CKD [22]. These findings suggest a critical role of RTN1A in renal tubular cell injury. However, the role of ER stress in hyperuricemia induced kidney injury hasn’t been studied.

The present study was designed to determine the expression of RTN1A and ER stress markers in the kidneys of HN rat model and human biopsies. We also liked to examine whether treatment of Febuxostat diminished ER stress and apoptosis of renal tubular cells and attenuated kidney injury in HN rat model. The study provides us a better understanding of ER stress in HN and a potential new therapeutic target for hyperuricemia induced kidney injury.

## RESULTS

### Expression of RTN1A and ER stress markers was increased in human kidney biopsies of HN patients

**Figure 1:**
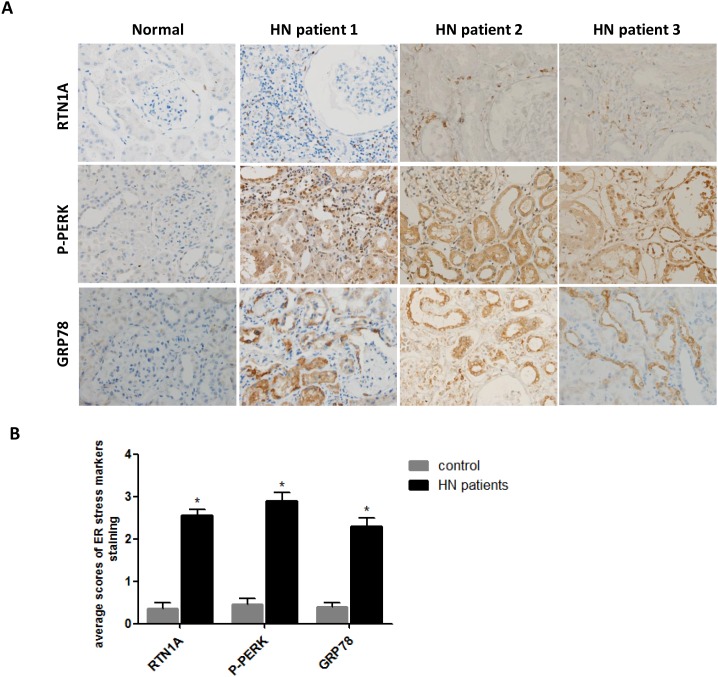
Expression of RTN1A and ER stress markers was increased in human kidney biopsies of HN patients **(A)** Immunostaining of RTN1A and ER stress markers in kidney sections of patients with HN. Representative images are shown for each group (Original magnification: ×400). **(B)** Semi-quantitative data of staining for RTN1A and ER stress markers in different groups of patients. Data are presented as means ±SEM. ^*^ *P*<0.05 vs. control group.

To examine whether ER stress was involved in HN, we determined the level of ER stress markers in the kidneys of hyperuricemia patients with kidney injury. We performed a retrospective review from year 2012 to 2017 and identified three CKD patients who underwent kidney biopsy and had a long history of hyperuricemia, recurrent attacks of gout and kidney stones detected by ultrasound. CKD was diagnosed based on KDOQI classification [23], with the exclusion of primarily glomerular nephritis, diabetic nephropathy and other secondary kidney diseases (Supplementary Table 1). Immunohistochemistry staining of ER stress markers was performed on paraffin embedded kidney sections from these three patients. Normal kidney section of nephrectomy sample was used as control. As shown in Supplementary Figure 1, tubular dilatation, interstitial fibrosis and focal infiltration of inflammatory cells were presented in the kidneys of HN patients. The intensity of the staining for RTN1A and ER stress markers (GRP78 and P-PERK) was significantly increased in kidney sections of hyperuricemia patients as compared to normal controls (Figure 1). The staining localized mostly in tubular compartment, which was consistent with the predominant tubular and interstitial injury in HN (Figure 1). These findings indicate that ER stress is associated with hyperuricemia induced kidney injury.

### Febuxostat attenuated kidney injury in hyperuricemic rats

Since we found ER stress was increased in hyperuricemia patients with kidney injury, we next examined the role of ER stress in a rat model of HN. Rats were gavaged with adenine (0.1 g/kg) and potassium oxonate (1.5 g/kg) daily for six weeks. 1 week after administration of adenine and potassium oxonate, HN rats were orally treated with Febuxostat (5mg/kg/day) or equal volumes of saline as vehicle for the following 5 weeks. As shown in Table 1, serum uric acid (SUA), serum creatinine(sCr), blood urea nitrogen(BUN), urine albumin/creatinine ratio (UACR), kidney/body weight ratio, fractional excretion of sodium (FENa) and fractional excretion of potassium (FEK) were all significantly increased while creatinine clearance rate (Ccr) was significantly reduced at 6 week after adenine and potassium oxonate administration in hyperuricemic rats when compared with the control rats receiving the vehicle (Table 1, Supplementary Table 4). Treatment of hyperuricemic rats with Febuxostat reduced proteinuria and improved renal function. In addition, hyperuricemic rats showed a significantly increased systolic blood pressure (SBP) which was also attenuated by Febuxostat treatment, while no difference in diastolic blood pressure (DBP) was observed between HN rat treated with or without Febuxostat (Table 1). These data suggest that hyperuricemia induced kidney injury and systolic hypertension could be reversed by Febuxostat treatment.

**Table 1:** Febuxostat attenuated kidney injury in hyperuricemic rats

Variables	Control	Control+Fx	HN	HN+Fx
ALB(g/L)	29.20±1.48	31.00±1.41	30.00±2.88	31.00±1.41
BUN(mmol/L)	5.78±0.34	5.98±0.5	13.18±2.48^**^	6.36±0.69^#^
Scr(μmol/l)	19.75±1.71	20.67±3.06	50.00±8.73^**^	25.29±2.06^#^
UA(umol/L)	57.00±6.75	55.33±3.21	311.67±13.05^**^	104.00±10.2^#^
ALT(u/L)	40.00±6.93	38.00±4.42	34.75±7.94	39.29±5.50
AST(u/L)	132.80±6.76	97.00±14.97	149.8±15.54	145.8±17.78
TC(mmol/L)	1.48±0.21	1.69±0.26	1.56±0.27	1.18±0.22
TG(mmol/L)	0.54±0.15	0.81±0.09	0.52±0.30	0.7±0.2
HDL(mmol/L)	0.72±0.11	0.82±0.14	0.98±0.16^*^	0.72±0.15^#^
LDL(mmol/L)	0.48±0.10	0.42±0.07	0.46±0.12	0.36±0.09
BG(mmol/L)	6.37±0.39	8.19±0.56	5.19±0.96	5.36±0.54
UACR(mg/mmol)	2.60±0.39	2.79±0.88	26.33±2.52^**^	5.38±1.27^#^
SBP(mmHg)	113.57±3.64	110.44±6.54	154.43±3.10^**^	136.75±6.49^*#^
Kidney/BW(mg/g)	2.78±0.18	2.95±0.05	5.57±0.49^**^	4.19±0.41^**#^
Ccr(ml/min/100g BW)	1.53±0.19	1.49±0.09	0.77±0.20^**^	1.13±0.20^*#^
FENa(%)	0.16±0.04	0.15±0.05	0.41±0.10^**^	0.19±0.04^#^
FEK(%)	10.9±1.07	11.5±0.95	32.1±1.52^**^	12.7±2.41^#^

Abbreviations: Fx: Febuxostat; HN: hyperuricemic nephropathy; BUN: blood urea nitrogen ; TC: total cholesterol ; TG: triglyceride; ALB: serum albumin; ALT: Alanine transaminase ; AST: aspertate aminotransferase ; HDL: high-density lipoprotein; LDL: low density lipoprotein ; BG: blood glucose ; UACR: urine albumin-to-creatinine ratio ; Ccr: creatinine clearance rate ; FENa: fractional excretion of sodium; FEK: fractional excretion of potassium; BW: Body Weight. ^*^p<0.05, ^**^p<0.01 for comparisons between the corresponding HN and control groups. ^#^ *p*<0.05 vs HN+Fx rats.

### Febuxostat ameliorated pathological injury and reduced uric acid crystals deposition in HN rat kidneys

**Figure 2:**
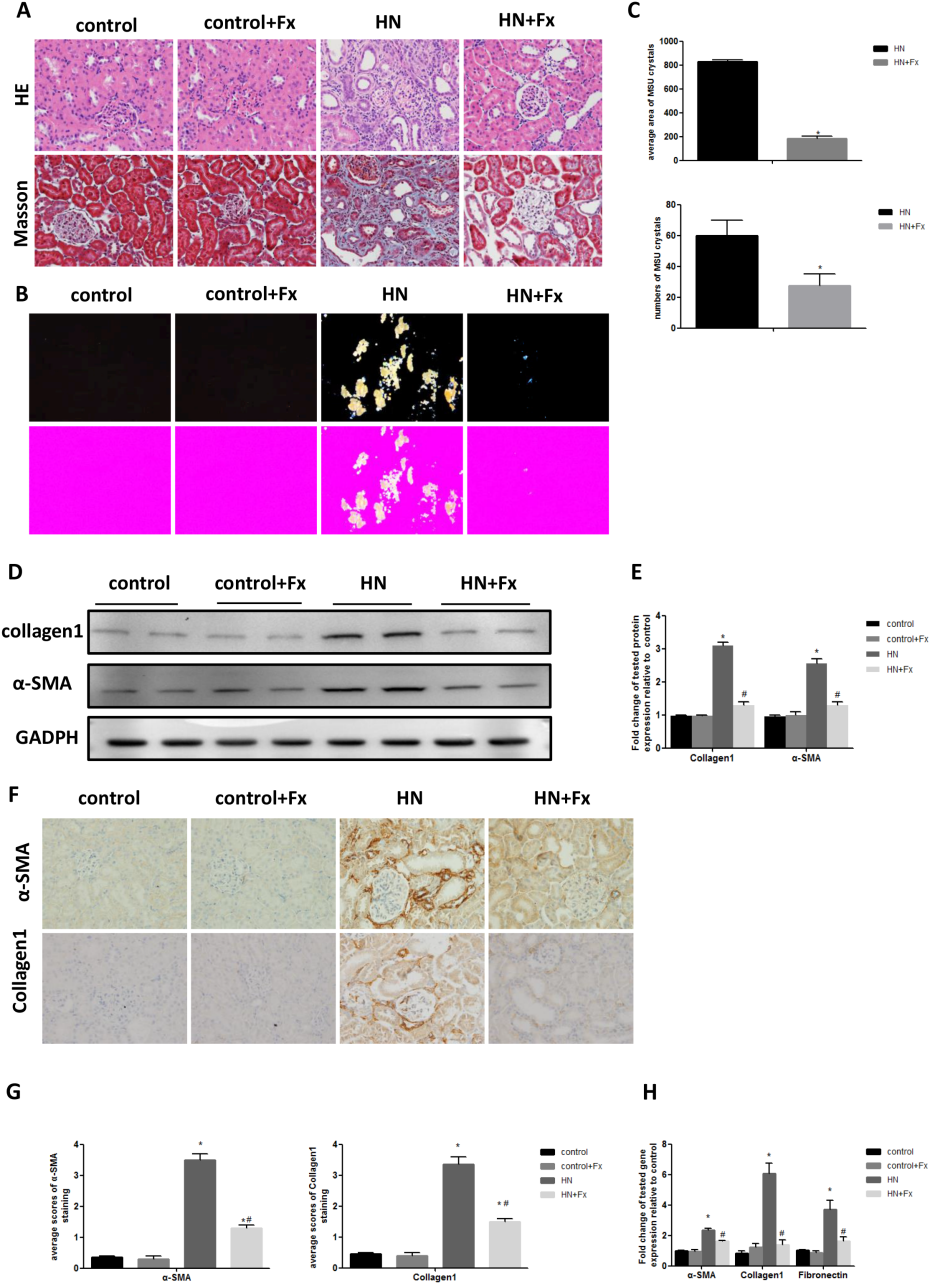
Febuxostat ameliorated pathological injury and reduced uric acid crystals deposition in HN rat kidneys **(A)** HE and Masson staining of kidney sections were compared between different groups of rats (Original magnification ×400). **(B)** The kidney sections under compensated polarized light showing anisotropic uric acid crystals in the kidney (Original magnification. ×200). **(C)** Semiquantitative analysis of urate crystals in the kidney section. **(D)** Western blot analysis of collagen1 and α-SMA in the kidney tissue lysates of hyperuricemic rats. **(E)** The densitometry analyses of western blots are shown. **(F)** Immunohistochemistry staining for collagen1 and α-SMA in kidneys and the representative pictures are shown (Original magnification x400). **(G)** Semi-quantitative data of collagen1 and α-SMA staining in different groups of rats. **(H)** Effect of Febuxostat on mRNA expression of fibrosis markers in the kidney tissue lysate. Data are presented as means ±SEM of four experiments. n = 8;^*^ *P*<0.05 vs. control group. ^#^ *P*<0.05 vs. HN+Fx group.

We then performed histological tests to examine the pathological changes in HN rat kidney. H&E and Masson’s trichrome staining showed that hyperuricemic rats developed significant tubular dilatation, focal infiltration of inflammatory cells and kidney fibrosis in interstitial compartment, which was markedly attenuated in Febuxostat treated HN rat. (Figure 2A, Supplementary Figure 4). Western blot and real-time PCR showed that the expression of fibrosis markers (α-SMA, collagen I and Fibronectin) were increased in HN rats as compared to normal control (Figure 2D, 2E, 2H). This was further confirmed by immunostaining of α-SMA and collagen I (Figure 2F, 2G). Febuxostat treatment reduced expression of fibrosis markers in HN rats, which is consistent with the histologic findings (Figure 2D-2H).

Since hyperuricemia contributes to the deposition of monosodium urate crystals (MUC) in tissues, such as joints and kidneys, we used compensated polarized light microscope to detect MUC deposition in the kidneys of hyperuricemic rats. Interestingly, HN rats showed large aggregated crystals in all the major areas of the kidney, particularly in the tubular compartment, which was significantly reduced by Febuxostat treatment (Figure 2B). Quantification data confirmed that the average number and area of MUC in HN rat kidneys were significantly reduced by the Febuxostat treatment (Figure 2C). These data indicate that treatment of Febuxostat not only attenuates renal tubular injury and fibrosis, but also reduces MUC deposition in HN rat kidneys.

### Febuxostat attenuated endoplasmic reticulum stress response in the kidney of hyperuricemic rats

**Figure 3:**
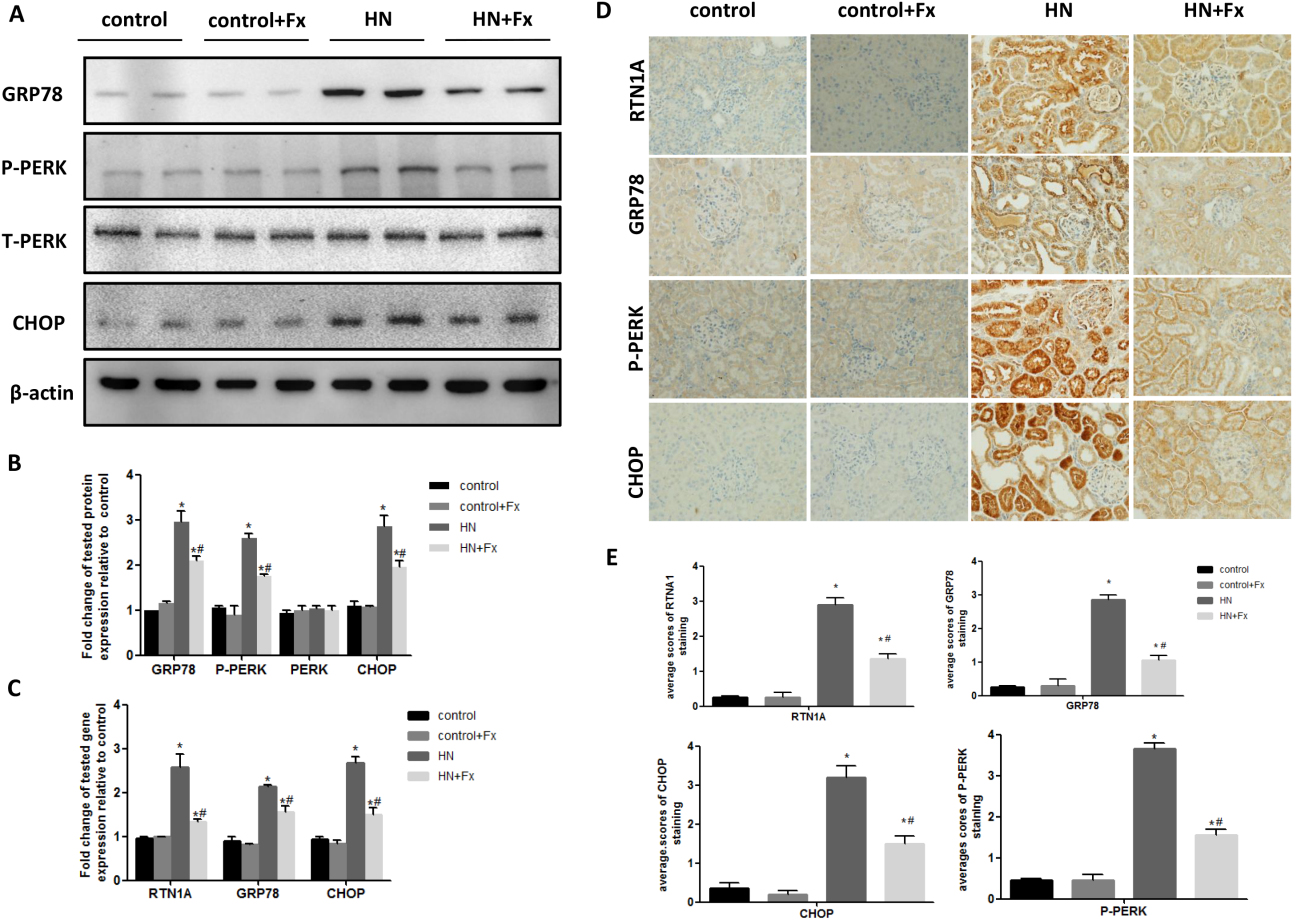
Febuxostat attenuated endoplasmic reticulum stress response in the kidney of hyperuricemic rats **(A)** Western blot analysis of ER stress markers in the kidney tissue lysates of hyperuricemic rats. **(B)** The densitometry analyses of western blots are shown. **(C)** Effect of Febuxostat on mRNA expression of ER stress markers in the kidney tissue lysates. **(D)** Immunohistochemistry staining for RTN1A and ER stress markers in kidneys and the representative pictures are shown (Original magnification x400). **(E)** Semi-quantitative data of RTN1A, p-PERK, GRP78 and CHOP staining in different groups of rats. Data are presented as means ±SEM of four experiments. n = 8;^*^ *P*<0.05 vs. control group. ^#^*P*<0.05 vs. HN+Fx group.

To clarify whether ER stress is involved in HN, we examined the expression of ER stress markers in the kidneys of hyperuricemic rats. The expression of RTN1A and ER stress markers GRP78, P-PERK and CHOP was significantly increased in the kidneys of hyperuricemic rats, as measured by either western blot or real-time PCR (Figure 3A-3C). This was also confirmed by immunostaining of RTN1A and other known ER stress markers (Figure 3D, 3E). In addition, expression of RTN1A, P-PERK and GRP78 was mainly localized in the tubular compartment of hyperuricemic rats, which was consistent with the tubulointerstitial damage in HN rats (Figure 3D). In HN rats treated with Febuxostat, the expression of ER stress markers was markedly suppressed at both protein and mRNA levels, suggesting a protective role of Febuxostat in HN via inhibition of RTN1A expression and ER stress response in kidney cells.

### Febuxostat reduced apoptosis of renal tubular cells in hyperuricemic rats

**Figure 4:**
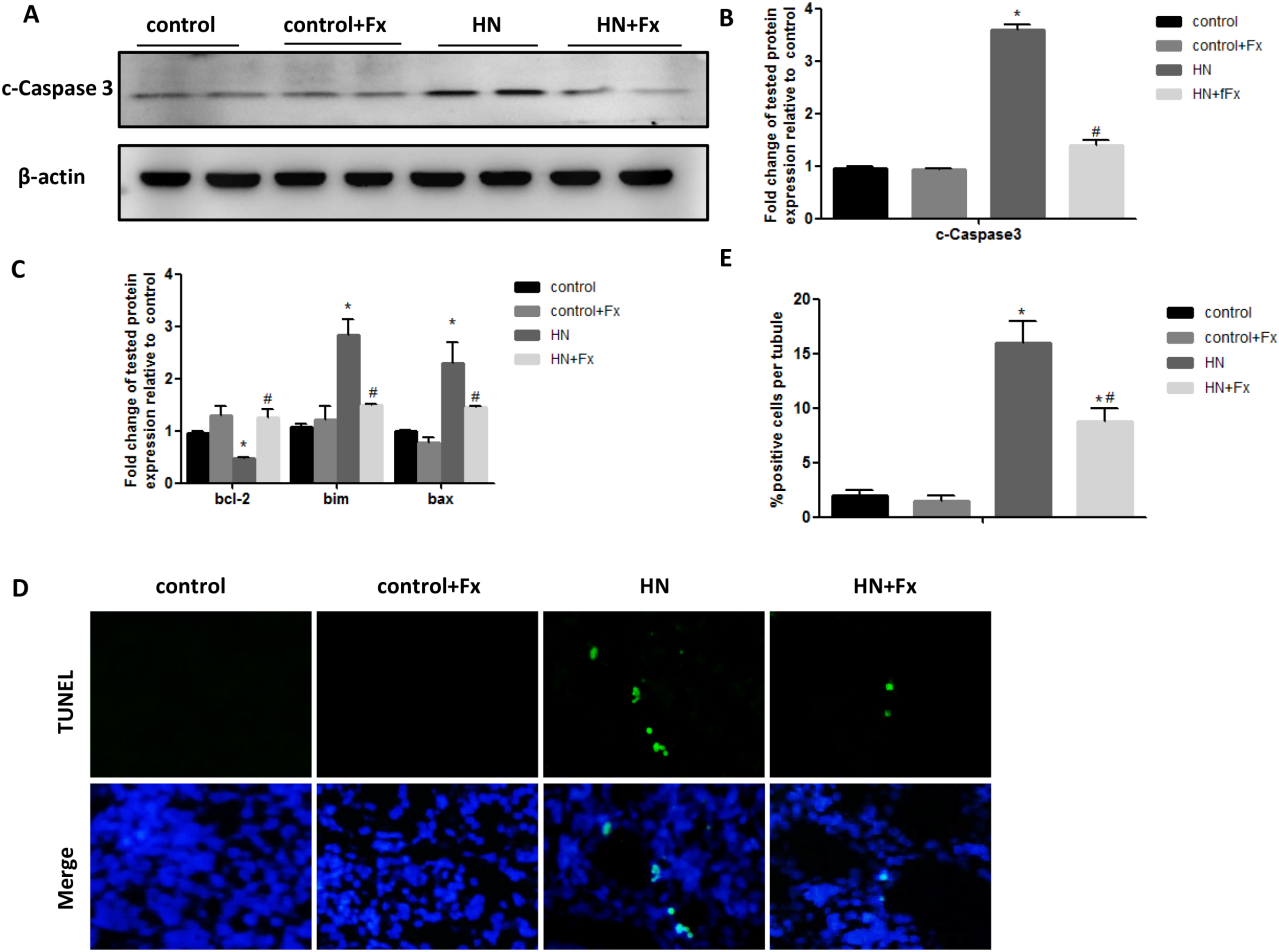
Febuxostat reduced apoptosis of renal tubular cells in hyperuricemic rats **(A)** Western blot analysis of c-caspase3 in the kidney tissue lysates of hyperuricemic rats. **(B)** The densitometry analyses of western blots for c-caspase3 are shown. **(C)** Expression of pro-apoptotic genes (bax, bcl-2, and bim) were measured by Real-time PCR. **(D)** TUNEL staining to measure the apoptosis of renal tubular epithelial cell in each rat group (Original magnification: ×200). **(E)** Quantitative analysis of the number of apoptotic cells in each rat group. Data are presented as means ±SEM of four groups. n = 8;^*^ *P*<0.05 vs. control group. ^#^ *P*<0.05 vs. HN+Fx group.

Since prolonged ER stress leads to apoptosis and our previous studies confirmed that ER stress contributed to apoptosis of renal tubular cells in animal models of proteinuric kidney diseases and acute kidney injury [21, 22], hereby we assessed whether ER stress was involved in the induction of apoptosis of kidney cells in HN rats. Western blot analysis of renal cortex revealed that the protein level of cleaved caspase-3 was significantly elevated in hyperuricemic rats (Figure 4A, 4B). The upregulation of pro-apoptotic genes (bim and bax-2) and down-regulation of anti-apoptotic gene bcl-2 were also found by real-time PCR (Figure 4C). Consistent with the inhibition of a sustained ER stress response, Febuxostat treatment also attenuated hyperuricemia-induced apoptosis in HN rat kidneys. We also detected an increase of apoptotic cells in kidney sections of hyperuricemic rats by TUNEL staining which was diminished by Febuxostat treatment (Figure 4D, 4E).

### Febuxostat reversed the expression of OAT1 and OAT3 and increased serum XOD activity in hyperuricemic rats

**Figure 5:**
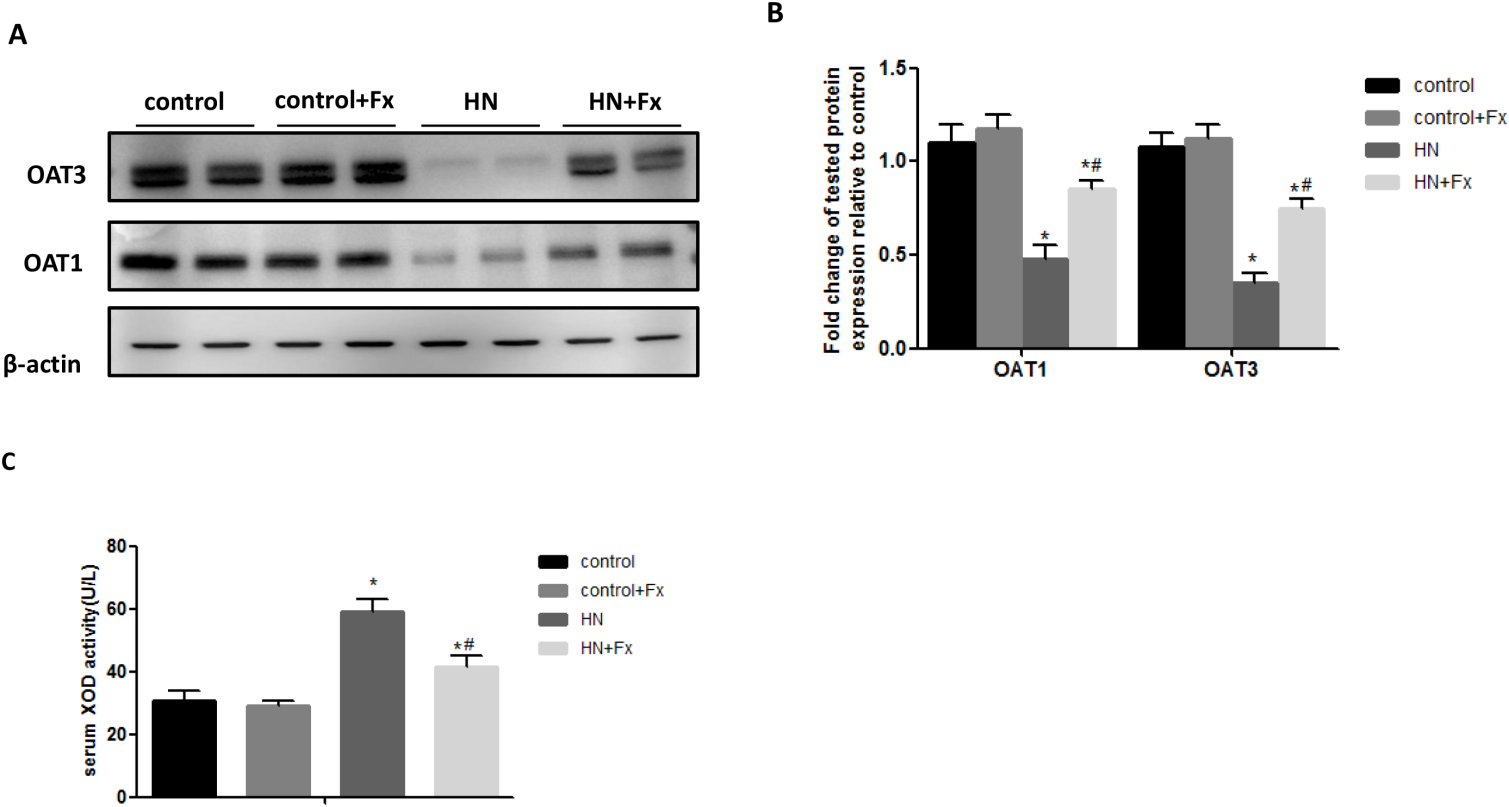
Febuxostat reversed the expression of OAT1 and OAT3 and increased serum XOD activity in hyperuricemic rats **(A)** Immunoblot analysis with specific antibodies against OAT1, OAT3 in the kidney tissue lysates of hyperuricemic rats. **(B)** The densitometry analyses of western blots are shown. **(C)** Serum XOD activity was examined by XOD kit. Data are presented as means ±SEM of four experiments. n = 8;^*^P<0.05 vs. control group. ^#^ *P*<0.05 vs. HN+Fx group.

Since OAT1 and OAT3 are two critical urate transporters located in renal proximal tubule cells mediating uric acid excretion, we tried to examine the expression of OAT1 and OAT3 in hyperuricemic rats. The expression of OAT1 and OAT3 were markedly reduced in HN rat kidneys when compared to normal controls as measured by western blot while Febuxostat treatment significantly increased OAT1 and OAT3 expression in the kidney of hyperuricemic rats (Figure 5A, 5B). It is known that hyperuricemia is associated with upregulation of serum XOD activity, we found that the activity of serum XOD was markedly increased in hyperuricemic rats, which was reversed by Febuxostat treatment (Figure 5C), suggesting that Febuxostat contributes to the regulation of uric transporter and suppression of the XOD activity in hyperuricemic rats.

### Febuxostat inhibited uric acid-induced expression of ER stress and pro-fibrosis markers in NRK-52E cells

**Figure 6:**
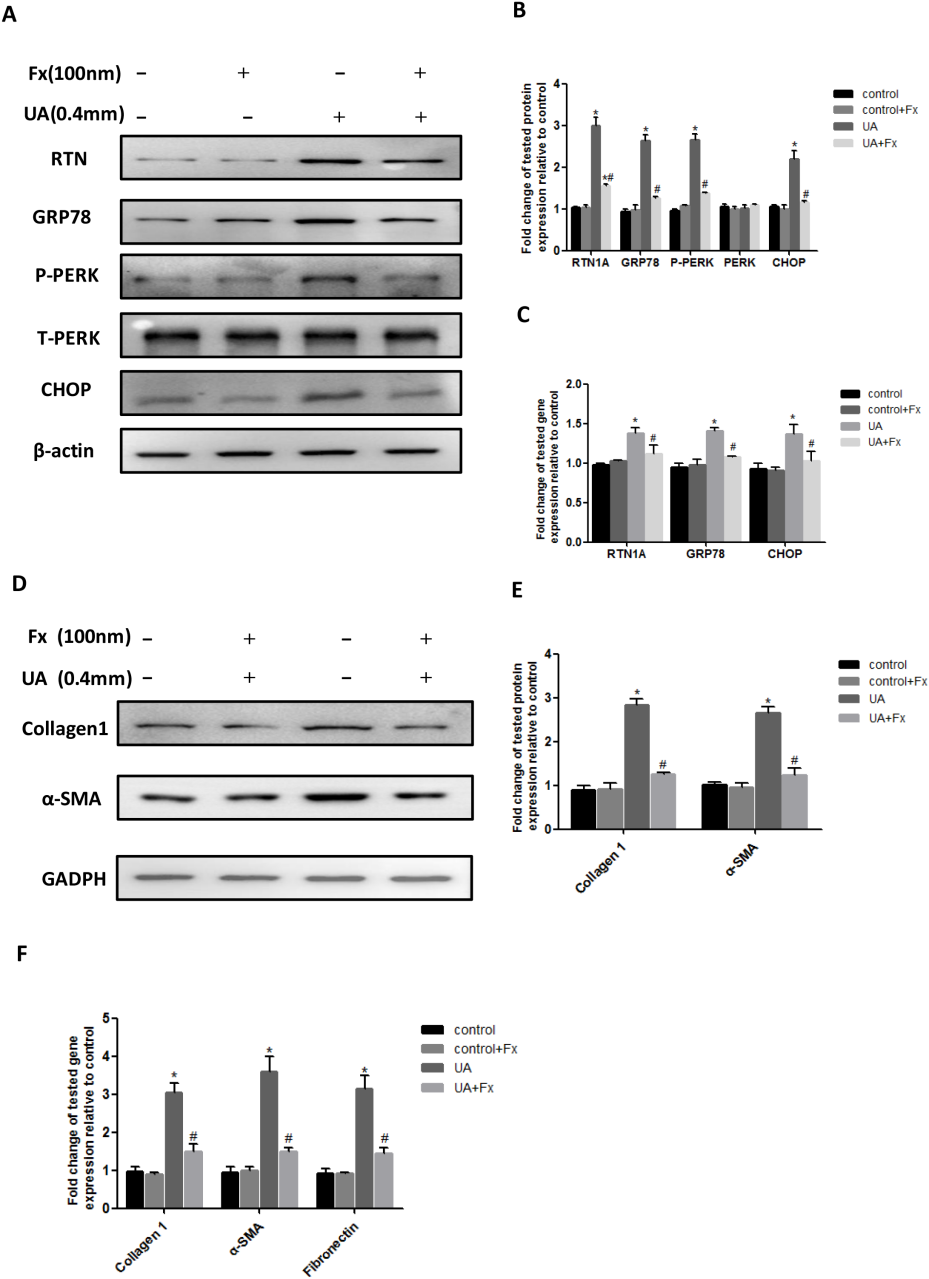
Febuxostat inhibited uric acid-induced expression of ER stress and pro-fibrosis markers in NRK-52E cells **(A)** Western blot analyses for RTN1A and ER stress markers. **(B)** Densitometric analysis of Western blots for RTN1A and ER stress markers. **(C)** Real-time PCR analyses for genes of RTN1A and ER stress markers (GRP78 and CHOP). **(D)** Western blot analyses for collagen 1 and α-SMA. **(E)** The densitometry analyses of western blots are shown. **(F)** Real-time PCR analyses for pro-fibrosis gene expression. ^*^p<0.05, compared to control group, ^#^ *p*<0.05, compared to UA+Fx group. Data are presented as means ±SEM of four experiments.

To further confirm the direct role of Febuxostat in the regulation of ER stress, we performed *in vitro* study in rat renal tubular epithelial cells (NRK-52E cells). Exposure of NRK-52E cells to uric acid at 0.1-0.4 mM for 24h resulted in an upregulation of the ER stress marker expression including GRP78, p-PERK and CHOP (Supplementary Figure 2A). In addition, the time course showed that expression of ER stress markers was significantly upregulated at 12h after uric acid stimulation and further increased in a time-dependent manner (Supplementary Figure 2B). Pretreatment with Febuxostat not only inhibited uric acid-induced expression of RTN1A and ER stress markers (GRP78, P-PERK and CHOP) (Figure 6A-6C), but also downregulated the fibrosis markers (Collagen 1, α-SMA and Fibronectin), as measured by western blot or real-time PCR(Figure 6D-6F). These findings confirmed that Febuxostat attenuated uric acid-induced ER stress and pro-fibrosis pathways in NRK-52E cells.

### Febuxostat treatment alleviated UA induced apoptosis of NRK-52E cells

**Figure 7:**
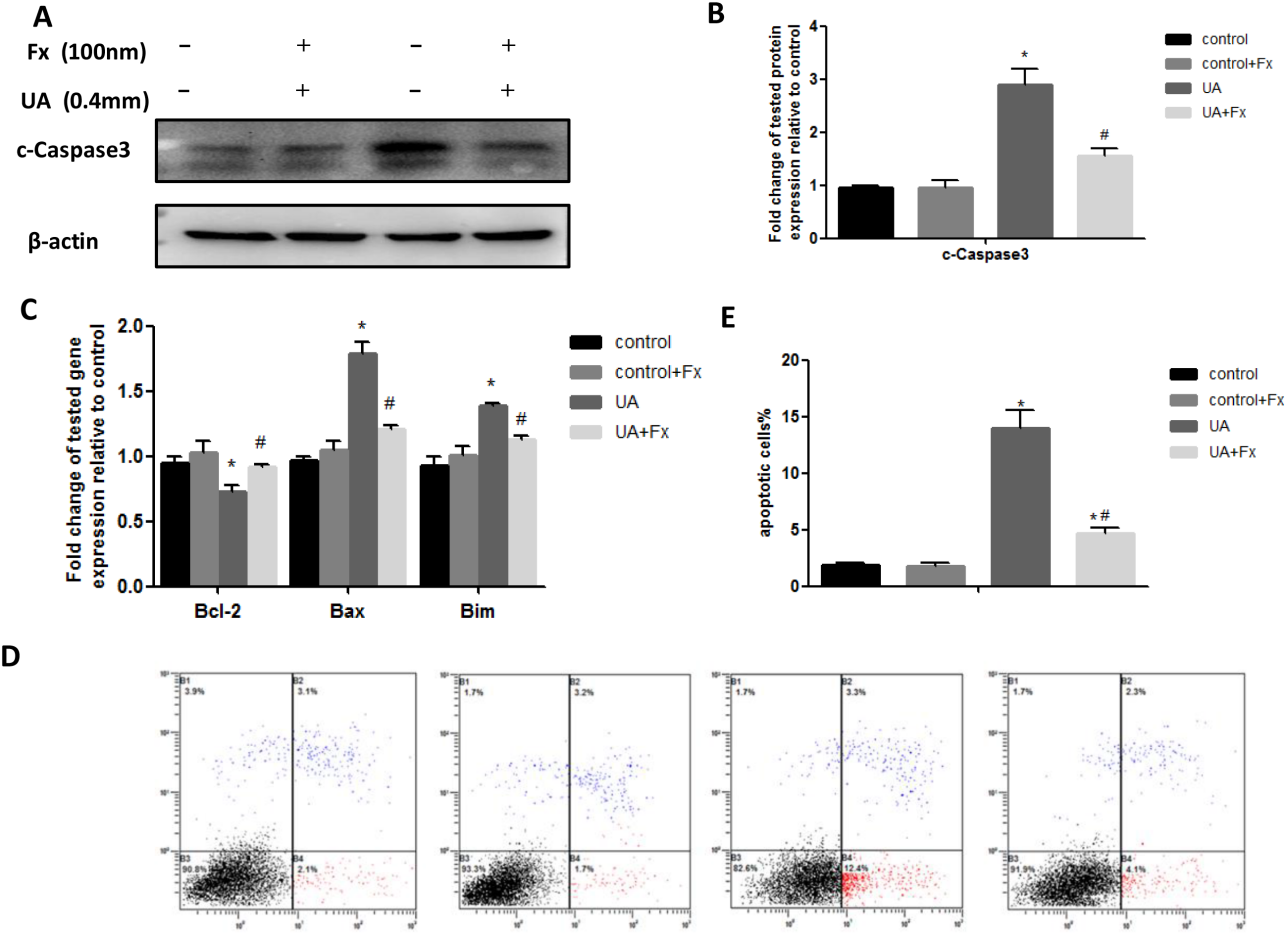
Febuxostat treatment alleviated UA induced apoptosis of NRK-52E cells **(A)** The kidney tissue lysates were subjected to Western blot analysis with specific antibodies against c-Caspase3. **(B)** Densitometric analysis of Western blots for c-Caspase3. **(C)** Apoptosis related markers were detected by real-time PCR. **(D)** Flow cytometry after double labeling with Annexin V and propidium iodide. **(E)** Quantification of apoptotic cells. Data are presented as means ±SEM of four experiments. n = 8;^*^ *P*<0.05 vs. control group. ^#^ *P*<0.05 vs. HN+Fx group.

Next, we determined whether Febuxostat inhibited uric acid-induced apoptosis of NRK-52E cells. We found that uric acid-induced apoptosis of NRK-52E cells was suppressed by Febuxostat treatment as assessed by western blot analysis of cleaved caspase-3 (Figure 7A, 7B) and real-time PCR analysis of apoptotic marker expression (bax, bcl-2, and bim) (Figure 7C). This finding was confirmed by flow cytometry analysis using Annexin V labeling (Figure 7D, 7E). Taken together, these data indicate that treatment with Febuxostat led to an inhibition of uric acid-induced apoptosis of NRK-52E cells.

## DISCUSSION

In the current study we examined whether Febuxostat had renal protective effects in HN rat kidneys. We used a classical HN rat model by administration of the mixture of adenine and potassium oxonate daily for 6 weeks. These rats exhibited persistent hyperuricemia, albuminuria and renal dysfunction, accompanied by reduced renal expression of urate transporters OAT1 and OAT3 and activation of serum XOD. Histologically, these hyperuricemic rats developed severe tubulointerstitial damage and fibrosis. We found that all these abnormalities were markedly improved after Febuxostat treatment, suggesting a protective role of Febuxostat in HN. Interestingly, we also found SBP was upregulated in HN rats which was normalized by Febuxostat treatment, consistent with the fact that hyperuricemia is a risk factor for hypertension [24] and the systolic blood pressure increases significantly in hyperuricemia patients [25]. In addition, a comparative study of Febuxostat and allopurinol in 141 cardiac surgery outpatients with hyperuricemia also showed that Febuxostat had an anti-hypertensive effect while this was not observed in the allopurinol-treated patients [26]. The previous studies suggest that activation of renin-angiotensin system (RAS) [3], reduction of endothelial nitric oxide levels and endothelial dysfunction [27, 28, 29] are potential mechanisms of hypertension in human and animal models of HN. Therefore, we speculated that the similar mechanisms are involved in the anti-hypertensive effect of Febuxostat in HN rats.

Accumulating evidences indicated hyperuricemia was a risk factor for CKD [30] and several mechanisms have been suggested including the uric acid induced epithelial-to-mesenchymal transition (EMT) of renal tubular cells [31], activation of TGF-β/Smad3 pathway [32] and increased renal oxidative stress [7]. A few of studies suggest an association of ER stress and uric acid-induced kidney cell injury. For example, uric acid-induced ER stress was found in rat glomerular mesangial cells [33]. In a human pathologic study from patients with UMOD gene mutation, who had familial juvenile hyperuricemic nephropathy, ER stress marker GRP78 was found to be highly expressed in renal tubular compartment [34], indicating a possible association between ER stress and UMOD related kidney disease. However, the exact role of ER stress in HN remains unclear. ER stress has been well recognized as one of the important mechanisms in the onset and progression of many kidney diseases, but the detailed mechanisms remain poorly understood [35, 36]. In recent studies, we identified reticulon-1A (RTN1A), an ER-associated protein reticulon-1, as a novel gene associated with the progression of kidney diseases. RTN1A-mediated ER stress and apoptosis contribute to tubular cell injury in diabetic nephropathy [16, 21], albumin overload-induced nephropathy [37] and AKI murine models [22].

Here, we confirmed a critical role of RTN1A and ER stress in uric acid induced tubular injury in cultured renal tubular cells and in human and animal model of HN. We confirmed that the expression of RTN1A and ER stress markers was increased in human kidney biopsy samples from HN patients. Consistent with the human data, expression of RTN1A and ER stress markers was also highly increased in the kidneys of HN rat. Treatment of Febuxostat lowered the uric acid level, reduced ER stress and apoptosis, and attenuated kidney injury in HN rats. In addition, we confirmed that uric acid-induced ER stress and apoptosis were also improved with Febuxostat treatment in cultured tubular cells. Our findings suggest that Febuxostat exerts its protective effect in HN most likely through inhibition of uric acid-induced ER stress. Further studies are required to confirm the role of RTN1A and ER stress in HN by using the knockout approach.

Our findings also suggest that Febuxostat reduces the aggregation of MUC and this might be another mechanism by which Febuxostat attenuated kidney injury. Polarized microscopy has been regarded as the “gold standard” to detect MSU crystals or tophus deposition in the joints of gout [38, 39]. However, few studies elaborated the fact on MUC crystals deposition in the kidney [40]. Hereby we used polarized light microscope and performed semi-quantitative measurement to examine hyperuricemia induced MUC deposition in the kidneys of HN rat. Notably, these HN rats exhibited massive monosodium urate (MSU) crystal accumulation in the kidneys, mostly in tubular compartment, which was remarkably reversed by Febuxostat treatment. It is known that the obstruction of the renal tubules with urate crystals is a major cause of renal injury [3].

Febuxostat has been widely used for the treatment of hyperuricemia and prevention of gout [41]. Recent studies reported the renal protective effects of Febuxostat including reduction of albuminuria, improvement of renal structural changes, and slowing down the progression of CKD [42]. Treatment with Febuxostat has also been shown to preserve renal function and prevent glomerular hypertension in 5/6 nephrectomized rats [43]. Multiple mechanisms have been suggested to contribute to the beneficial effects of Febuxostat in the diabetic kidney such as anti-inflammation, alleviation of oxidative stress, and inhibition of pro-fibrotic signaling [44, 45]. Febuxostat also exhibited a protective role in AKI experimental model through activation of BMP-7 signaling and inhibition of USAG-1 expression in unilateral ureteral obstruction (UUO) rats [45]. Here, we confirmed that Febuxostat attenuated hyperuricemia-induced kidney injury in HN rats, suggesting a potential therapeutic role of Febuxostat in patients with hyperurecemia-related CKD.

In conclusion, we reported here that expression of RTN1A and ER stress marker was increased in rats and human with HN. Febuxostat, a novel non–purine analogue inhibitor of xanthine oxidase, improves systolic hypertension and kidney tubulointerstitial injury in HN rats likely through attenuation of ER stress and MUC crystal deposition in tubules. Febuxostat also suppressed uric acid-induced ER stress and apoptosis in cultured tubular cells. This study suggests a therapeutic role of Febuxostat in hyperurecemic-related CKD. Further study is required to explore whether inhibition of ER stress could be a new therapy for hyperuricemia-induced kidney injury.

## MATERIALS AND METHODS

### Animal models

Male Sprague-Dawley rats weighing 180g-200g were purchased from the National Mode Animal Centre of Nanjing University (Nanjing, China) and housed under a constant 12-hour light–dark cycle at a temperature between 21°C and 23 °C and allowed free access to food and water. All the animal experiments were agreed by the Animal Care and Ethical Committee of Sixth People’s Hospital Affiliated to Shanghai Jiaotong University. The rats were randomly divided into four groups (n = 8/each group), both control and HN rats treated with Febuxostat (5mg/kg/day) or equal volumes of saline. HN rat model was induced by orally feeding with the mixture of adenine (0.1 g/kg/day) and potassium oxonate (1.5 g/kg/day) daily for six weeks. 1 week after administration of adenine and potassium oxonate, Febuxostat or equal volumes of saline was gavaged for the next 5 weeks. The animals were sacrificed after 6 weeks.

### Cell culture and treatment

NRK-52E cells (ATCC, Manassas, VA) were cultured in DMEM (Sigma-Aldrich) containing 10% FBS, 1%penicillin, and streptomycin in an atmosphere of 5% CO2 and 95% air at 37°C. NRK-52E cells were starved with 0.5% FBS for 12 hours and then exposed to uric acid (Sigma-Aldrich) (0.4 mM) with or without the pretreatment of febuxostat. Cells were harvested for immunoblot analysis, real-time PCR and FITC Annexin V apoptosis detection.

### Measurement of blood and urine biochemistry indices

Serum uric acid, creatinine, blood urea nitrogen and other biochemistry indices were detected by an automatic biochemistry analyzer (Hitachi Model 7600, Japan).

### Serum xanthine oxidase (XOD) activity assessment

Serum XOD activity was tested using the assay kits (Jiancheng, Nanjing, China) according to the protocol provided by the manufacturer.

### Blood pressure assessment

Blood pressure was measured using the noninvasive tail cuff blood pressure system (ALC-NIBP, Shanghai, China). Rats were accustomed to the tail cuff manometer and restraining device five times per week for 2 weeks before the day of actual data acquisition.

### Histology and morphometry analysis

Tissues either embedded in paraffin or frozen in OCT compound, then sectioned to 3μm thickness for light or polarized light microscopy. Hematoxylin-eosin (HE) and Masson trichrome staining were performed to assess histological injury and fibrosis. Frozen sections of kidney were applied to measure anisotropism of uric acid crystals under polarized light microscope. Quantification of MUC was carried out using Image J software. A total of 20 views per slide were randomly obtained with a digital camera at 200 x magnification. Crystal numbers and area were counted.

### Immunohistochemistry staining analysis

Formalin-fixed kidneys were embedded in paraffin. Kidney sections were stained using the following antibodies: RTN1A, GRP78, CHOP, α-SMA, Collegen1 (Abcam, USA) and P-PERK (Santa Cruz, CA). The staining of glomerulus and tubulointerstitium was semi-quantitatively scored separately on a scale of 0–4 in a blinded manner by two independent researchers as previously described [21].

### Western blot analysis

Tissue or cells were lysed with RIPA buffer containing protease and phosphatase inhibitor cocktail. The specific antibodies were used for immunoblot analysis (Supplementary Table 2). We repeated each Western blot analysis using protein from three different and separate experiments. The specific protein bands were scanned using Western Blotting Detection System (BIO-RAD).

### Real-time PCR quantitation

Total RNA was extracted from the renal cortex sample using Trizol (Invitrogen, Carlsbad, CA, USA). RNA was reversed-transcribed into c-DNA using the Superscript III First-Strand Synthesis Super Mix (Invitrogen, Carlsbad, CA, USA). Real-time PCR was performed with the StepOne Plus System (Applied Biosystems, Foster City, CA, USA) using SYBR Green Master Mix (Qiagen). Using the 2−ΔΔCt method, relative gene expression levels were determined (Supplementary Table 3). Gene expression was normalized to GAPDH.

### Apoptosis assessment

Apoptotic cells were measured on kidney frozen sections using an *in situ* cell death detection kit (Roche Diagnostics, Mannheim, Germany). Apoptotic cells with nuclei staining green fluorescence were calculated using fluorescent microscopy. Ten fields per slide were quantified for apoptotic nuclei.

FITC Annexin V Apoptosis Detection Kit (BD Co Ltd) was used to measure the apoptosis rate of NRK-52E cells. The number of cells labeled with propidium iodide and Annexin V-FITC was tested with the flow cytometer (Beckman Coulter, Beckman Coulter Inc, California, USA). The data were assessed using CellQuest software (BD Biosciences).

### Statistical analysis

All the data were analyzed by SPSS software 19.0 (IBM, Armonk, NY, USA). They are showed as mean ± SEM. One way ANOVA followed by Bonferroni correction was used to compare the data of more than two groups while two-sided unpaired t test was used for non-parametric data comparison. A value of *P* < 0.05 was determined statistically significant.

## SUPPLEMENTARY MATERIALS FIGURES AND TABLES



## References

[1] Lin B, Shao L, Luo Q, Ou-yang L, Zhou F, Du B, He Q, Wu J, Xu N, Chen J (2014). Prevalence of chronic kidney disease and its association with metabolic diseases: a cross-sectional survey in Zhejiang province, Eastern China. BMC Nephrol.

[2] Sanchez-Lozada LG, Tapia E, Lopez-Molina R, Nepomuceno T, Soto V, Avila-Casado C, Nakagawa T, Johnson RJ, Herrera-Acosta J, Franco M (2007). Effects of acute and chronic L-arginine treatment in experimental hyperuricemia. Am J Physiol Renal Physiol.

[3] Mazzali M, Hughes J, Kim YG, Jefferson JA, Kang DH, Gordon KL, Lan HY, Kivlighn S, Johnson RJ (2001). Elevated uric acid increases blood pressure in the rat by a novel crystal-independent mechanism. Hypertension.

[4] Long CL, Qin XC, Pan ZY, Chen K, Zhang YF, Cui WY, Liu GS, Wang H (2008). Activation of ATP-sensitive potassium channels protects vascular endothelial cells from hypertension and renal injury induced by hyperuricemia. J Hypertens.

[5] Gude D, Chennamsetty S, Jha R (2013). Fathoming uric acid nephropathy. Saudi J Kidney Dis Transpl.

[6] Wang Y, Bao X (2015). Retraction note: effects of uric acid on endothelial dysfunction in early chronic kidney disease and its mechanisms. Eur J Med Res.

[7] Cristobal-Garcia M, Garcia-Arroyo FE, Tapia E, Osorio H, Arellano-Buendia AS, Madero M, Rodriguez-Iturbe B, Pedraza-Chaverri J, Correa F, Zazueta C, Johnson RJ, Lozada LG (2015). Renal oxidative stress induced by long-term hyperuricemia alters mitochondrial function and maintains systemic hypertension. Oxid Med Cell Longev.

[8] Okamoto K, Eger BT, Nishino T, Kondo S, Pai EF, Nishino T (2003). An extremely potent inhibitor of xanthine oxidoreductase. Crystal structure of the enzyme-inhibitor complex and mechanism of inhibition. J Biol Chem.

[9] Burns CM, Wortmann RL (2011). Gout therapeutics: new drugs for an old disease. Lancet.

[10] Febuxostat 40 mg and 80 mg NDA No. 21–856 Indication: treatment of hyperuricemia in patients with gout. Briefing document for Advisory Committee. Division of Anesthesia, Analgesia, and Rheumatology Products FDA Advisory Committee Meeting, 24 November 2008. http://www.fda.gov/ohrms/dockets/ac/08/briefing/2008-4387b1-02-Takeda

[11] Becker MA, Schumacher HJ, Wortmann RL, MacDonald PA, Eustace D, Palo WA, Streit J, Joseph-Ridge N (2005). Febuxostat compared with allopurinol in patients with hyperuricemia and gout. N Engl J Med.

[12] Quilis N, Andres M, Gil S, Ranieri L, Vela P, Pascual E (2016). Febuxostat for patients with gout and severe chronic kidney disease: which is the appropriate dosage? Comment on the Article by Saag et al. Arthritis Rheumatol.

[13] Richette P, Perez-Ruiz F, Doherty M, Jansen TL, Nuki G, Pascual E, Punzi L, So AK, Bardin T (2014). Improving cardiovascular and renal outcomes in gout: what should we target?. Nat Rev Rheumatol.

[14] Susztak K, Raff AC, Schiffer M, Bottinger EP (2006). Glucose-induced reactive oxygen species cause apoptosis of podocytes and podocyte depletion at the onset of diabetic nephropathy. Diabetes.

[15] Yu L, Li S, Tang X, Li Z, Zhang J, Xue X, Han J, Liu Y, Zhang Y, Zhang Y, Xu Y, Yang Y, Wang H (2017). Diallyl trisulfide ameliorates myocardial ischemia-reperfusion injury by reducing oxidative stress and endoplasmic reticulum stress-mediated apoptosis in type 1 diabetic rats: role of SIRT1 activation. Apoptosis.

[16] Fan Y, Zhang J, Xiao W, Lee K, Li Z, Wen J, He L, Gui D, Xue R, Jian G, Sheng X, He JC, Wang N (2017). Rtn1a-mediated Endoplasmic Reticulum Stress in podocyte injury and diabetic nephropathy. Sci Rep.

[17] Cybulsky AV (2013). The intersecting roles of endoplasmic reticulum stress, ubiquitin- proteasome system, and autophagy in the pathogenesis of proteinuric kidney disease. Kidney Int.

[18] Yum V, Carlisle RE, Lu C, Brimble E, Chahal J, Upagupta C, Ask K, Dickhout JG (2017). Endoplasmic reticulum stress inhibition limits the progression of chronic kidney disease in the Dahl salt-sensitive rat. Am J Physiol Renal Physiol.

[19] Cybulsky AV, Takano T, Papillon J, Bijian K (2005). Role of the endoplasmic reticulum unfolded protein response in glomerular epithelial cell injury. J Biol Chem.

[20] Nakajo A, Khoshnoodi J, Takenaka H, Hagiwara E, Watanabe T, Kawakami H, Kurayama R, Sekine Y, Bessho F, Takahashi S, Swiatecka-Urban A, Tryggvason K, Yan K (2007). Mizoribine corrects defective nephrin biogenesis by restoring intracellular energy balance. J Am Soc Nephrol.

[21] Fan Y, Xiao W, Li Z, Li X, Chuang PY, Jim B, Zhang W, Wei C, Wang N, Jia W, Xiong H, Lee K, He JC (2015). RTN1 mediates progression of kidney disease by inducing ER stress. Nat Commun.

[22] Fan Y, Xiao W, Lee K, Salem F, Wen J, He L, Zhang J, Fei Y, Cheng D, Bao H, Liu Y, Lin F, Jiang G (2017). Inhibition of reticulon-1A-mediated endoplasmic reticulum stress in early AKI attenuates renal fibrosis development. J Am Soc Nephrol.

[23] Inker LA, Astor BC, Fox CH, Isakova T, Lash JP, Peralta CA, Kurella TM, Feldman HI (2014). KDOQI US commentary on the 2012 KDIGO clinical practice guideline for the evaluation and management of CKD.. Am J Kidney Dis.

[24] Mazza A, Lenti S, Schiavon L, Monte AD, Townsend DM, Ramazzina E, Rubello D, Casiglia E (2017). Asymptomatic hyperuricemia is a strong risk factor for resistant hypertension in elderly subjects from general population. Biomed Pharmacother.

[25] Avula NR, Shenoy D (2016). Evaluation of association of hyperuricaemia with metabolic syndrome and insulin resistance. J Clin Diagn Res.

[26] Sezai A, Soma M, Nakata K, Hata M, Yoshitake I, Wakui S, Hata H, Shiono M (2013). Comparison of febuxostat and allopurinol for hyperuricemia in cardiac surgery patients (NU-FLASH Trial). Circ J.

[27] Johnson RJ, Segal MS, Srinivas T, Ejaz A, Mu W, Roncal C, Sanchez-Lozada LG, Gersch M, Rodriguez-Iturbe B, Kang DH, Acosta JH (2005). Essential hypertension, progressive renal disease, and uric acid: a pathogenetic link?. J Am Soc Nephrol.

[28] Feig DI, Soletsky B, Johnson RJ (2008). Effect of allopurinol on blood pressure of adolescents with newly diagnosed essential hypertension: a randomized trial. JAMA.

[29] Shirakura T, Nomura J, Matsui C, Kobayashi T, Tamura M, Masuzaki H (2016). Febuxostat, a novel xanthine oxidoreductase inhibitor, improves hypertension and endothelial dysfunction in spontaneously hypertensive rats. Naunyn Schmiedebergs Arch Pharmacol.

[30] Yang Z, Xiaohua W, Lei J, Ruoyun T, Mingxia X, Weichun H, Li F, Ping W, Junwei Y (2010). Uric acid increases fibronectin synthesis through upregulation of lysyl oxidase expression in rat renal tubular epithelial cells. Am J Physiol Renal Physiol.

[31] Ryu ES, Kim MJ, Shin HS, Jang YH, Choi HS, Jo I, Johnson RJ, Kang DH (2013). Uric acid-induced phenotypic transition of renal tubular cells as a novel mechanism of chronic kidney disease. Am J Physiol Renal Physiol.

[32] Liu N, Wang L, Yang T, Xiong C, Xu L, Shi Y, Bao W, Chin YE, Cheng SB, Yan H, Qiu A, Zhuang S (2015). EGF receptor inhibition alleviates hyperuricemic nephropathy. J Am Soc Nephrol.

[33] Li S, Zhao F, Cheng S, Wang X, Hao Y (2013). Uric acid-induced endoplasmic reticulum stress triggers phenotypic change in rat glomerular mesangial cells. Nephrology (Carlton).

[34] Adam J, Bollee G, Fougeray S, Noel LH, Antignac C, Knebelman B, Pallet N (2012). Endoplasmic reticulum stress in UMOD-related kidney disease: a human pathologic study. Am J Kidney Dis.

[35] Chiang CK, Hsu SP, Wu CT, Huang JW, Cheng HT, Chang YW, Hung KY, Wu KD, Liu SH (2011). Endoplasmic reticulum stress implicated in the development of renal fibrosis. Mol Med.

[36] Noh MR, Kim JI, Han SJ, Lee TJ, Park KM (2015). C/EBP homologous protein (CHOP) gene deficiency attenuates renal ischemia/reperfusion injury in mice. Biochim Biophys Acta.

[37] Xiao W, Fan Y, Wang N, Chuang PY, Lee K, He JC (2016). Knockdown of RTN1A attenuates ER stress and kidney injury in albumin overload-induced nephropathy. Am J Physiol Renal Physiol.

[38] Zhang W, Doherty M, Bardin T, Pascual E, Barskova V, Conaghan P, Gerster J, Jacobs J, Leeb B, Liote F, McCarthy G, Netter P, Nuki G (2006). EULAR evidence based recommendations for gout. Part II: management. Report of a task force of the EULAR Standing Committee for International Clinical Studies Including Therapeutics (ESCISIT). Ann Rheum Dis.

[39] Richette P, Bardin T (2010). Gout. Lancet.

[40] Preitner F, Pimentel A, Metref S, Berthonneche C, Sarre A, Moret C, Rotman S, Centeno G, Firsov D, Thorens B (2015). No development of hypertension in the hyperuricemic liver-Glut9 knockout mouse. Kidney Int.

[41] Pascual E, Andres M, Vazquez-Mellado J, Dalbeth N (2016). Severe gout: strategies and innovations for effective management. Joint Bone Spine.

[42] Komers R, Xu B, Schneider J, Oyama TT (2016). Effects of xanthine oxidase inhibition with febuxostat on the development of nephropathy in experimental type 2 diabetes. Br J Pharmacol.

[43] Sanchez-Lozada LG, Tapia E, Soto V, Avila-Casado C, Franco M, Wessale JL, Zhao L, Johnson RJ (2008). Effect of febuxostat on the progression of renal disease in 5/6 nephrectomy rats with and without hyperuricemia. Nephron Physiol.

[44] Lee HJ, Jeong KH, Kim YG, Moon JY, Lee SH, Ihm CG, Sung JY, Lee TW (2014). Febuxostat ameliorates diabetic renal injury in a streptozotocin-induced diabetic rat model. Am J Nephrol.

[45] Cao J, Li Y, Peng Y, Zhang Y, Li H, Li R, Xia A (2015). Febuxostat prevents renal interstitial fibrosis by the activation of BMP-7 signaling and inhibition of USAG-1 expression in Rats. Am J Nephrol.

