# Supplementary material for: Febuxostat attenuates ER stress mediated kidney injury in a rat model of hyperuricemic nephropathy
Source: Oncotarget. 2017 Nov 30;8(67):111295–308. doi: 10.18632/oncotarget.22784 (PMC5762322; doi:10.18632/oncotarget.22784)
Supplement: Supplementary file 1 [file oncotarget-08-111295-s001.pdf]

Febuxostat attenuates ER stress mediated kidney injury in a rat model of hyperuricemic nephropathy

SUPPLEMENTARY MATERIALS

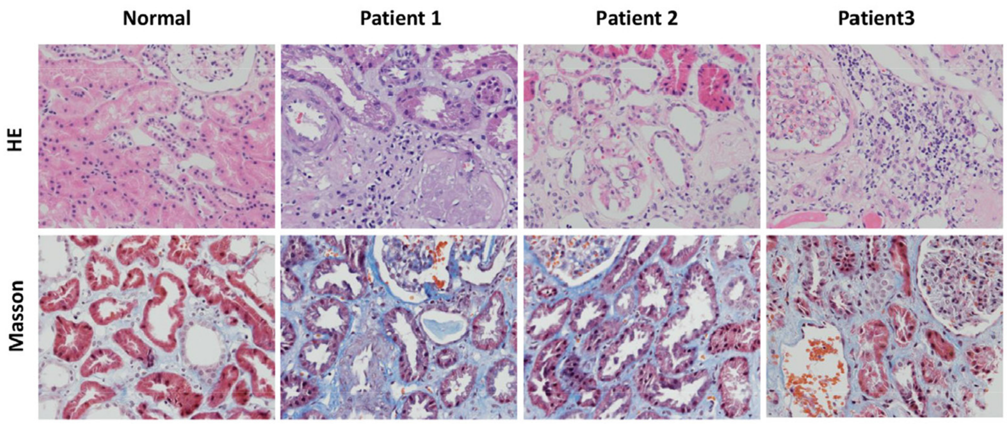

Supplementary Figure 1: Renal pathological changes in patients with hyperuricemia related kidney injury. Representative HE and Masson stained images of kidney biopsy sections in patients with hyperuricemia related kidney injury. (original magnification, ×400).

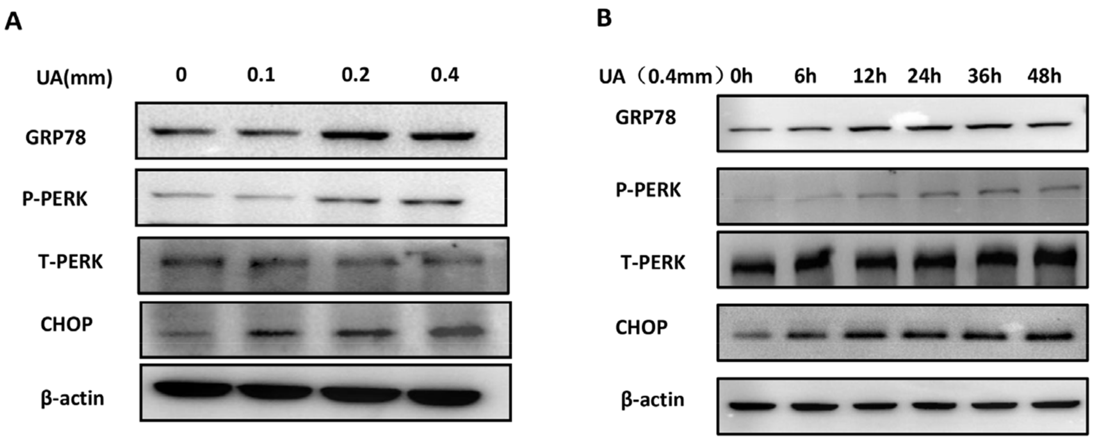

Supplementary Figure 2: ER stress was increased in uric acid induced NRK-52E cells. **(A)** Dose-dependent effect of uric acid on the expression of ER stress markers in NRK-52E cells. **(B)** Time-dependent effect of uric acid on the expression of ER stress markers in NRK-52E cells. Data are presented as means ±SEM of four experiments. n = 8;* *P*<0.05 vs. control group. # *P*<0.05 vs. HN+Fx group.

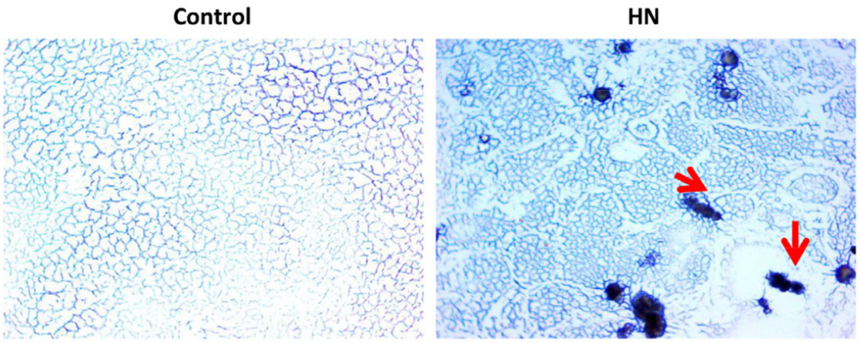

Supplementary Figure 3: Deposition of MSU crystals in the kidney of HN rats. The kidney sections under compensated polarized light showied monosodium urate (MSU) crystals located mostly in the renal tubulointerstitial compartment (original magnification, ×200).

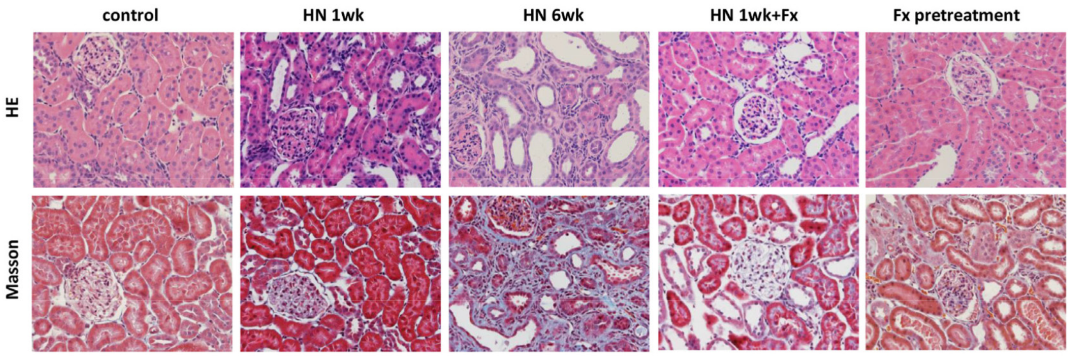

Supplementary Figure 4: Comparison of Renal histological change among HN rats and Febuxostat treated groups at different time points. HE and Masson staining of paraffin embedded kidney sections (original magnification, ×400).

Supplementary Table 1: The clinical characteristics of the patients with hyperuricemia induced kidney injury

HN patients	Patient 1	Patient 2	Patient 3
Gender	male	male	female
Age(year)	19	43	26
The history of Hyperuricemia (year)	5	10	4
The history of gout(year)	3	10	4
Complications			
primary glomerulonephritis(year)	none	none	none
Hypertension history(year)	1	7	none
Diabetes history(year)	none	none	none
Others(year)	none	none	none
The frequency of joint pain	Heel pain (3-4 times /year)	knee pain (intermittent attack)	Feet pain (3-4 times/year)
Renal biopsy report	Tubulointerstitial fibrosis with inflammatory cells infiltrated in the nephric tubules, renal tubular atrophy	tubulointerstitial fibrosis with inflammatory cells infiltrated in the nephric tubules, renal tubular atrophy	tubulointerstitial focal fibrosis and edema with inflammatory cells infiltrated in the nephric tubules
Medicine administration	Benzbromarone(3 year)	Benzbromarone(10year)	Allopurinol(4 year)
eGFR(mL/min/1.73m^2)	70.86	49.6	45.88
Proteinuria (mg/d)	0.43	0.36	0.48
24h urine uric acid(umol/24h)	2920	4356	3980
UA(umol/L)	551	499	556
sCr(μmol/L)	120	136	134
BUN(mmol/L)	3.8	8.9	6.1
ALT(U/L)	15	34	36
AST(U/L)	19	17	26
TC(mmol/L)	4.16	7.55	5.05
TG(mmol/L)	1.67	3.46	1.61
HDL(mmol/L)	0.79	2.38	1.3
LDL(mmol/L)	2.75	3.41	3.21
BG(mmol/L)	5.19	5.5	5.44
Kidney stones/location. numbers(by ultrasound)	Stones/left/1	Stones/ bilateral/6	Stones/left/1

Supplementary Table 2: Antibodies used in western blot

Antibodies	Company	Cat. No	Species	Molecular Weight
RTN1A	ABCAM	ab8957	Mouse	135ka
GRP78	ABCAM	ab21685	Rabbit	78ka
α-SMA	ABCAM	ab5694	Rabbit	42ka
Collagen1	ABCAM	Ab34710	Rabbit	139ka
CHOP	Cell Signaling	#2895	Mouse	27ka
P-PERK	Santa Cruz	sc-32577	Rabbit	125ka
OAT1	SIGMA-ALDRICH	SAB2108714	Rabbit	62ka
OAT3	Santa Cruz	Sc-293 264	Mouse	62ka
C-caspase3	Cell Signaling	#9664	Rabbit	17ka

Supplementary Table 3: Primer sequences used in real-time PCR

Target gene	Primer sequences
RTN1A	Forward 5' AAGGGGTCCATGCACCTAAC 3'<br>Reverse 5' GTCCGATTTCCGTCAGTGTG 3'
Bax	Forward 5' GGCGATGAACTGGACAACAA 3'
	Reverse 5' GCAAAGTAGAAAAGGGCAACC 3'
GRP78	Forward 5' GACTGGAATCCCTCCTGCTC 3'
	Reverse 5' GGTCAGGCGGTTTTGGTC 3'
CHOP	Forward 5' CACAAGCACCTCCCAAAGC 3'
	Reverse 5' CTCTCATTCTCCTGCTCCTTCTC 3'
CollagenI	Forward 5' ATCCTGCCGATGTCGCTAT 3'
	Reverse 5' CCACAAGCGTGCTGTAGGT 3'
Fibronectin	Forward 5' CACACCTGTGACCAGCAACAC 3'
	Reverse 5' T CATCTCCTTCCTCGCTCAGTTC 3'
α-SMA	Forward 5' ATTCCTTCGTGACTACTGCTGAG 3'
	Reverse 5' CCCATCAGGCAGTTCGTAGC 3'
Bcl-2	Forward 5' GGGAGCGTCAACAGGGAG 3'
	Reverse 5' ACAGCCAGGAGAAATCAAACAG 3'
Bim	Forward 5' AAGGCAGTCTCAGGAGGAACC 3'
	Reverse 5' ACGCCCTCCTCGTGTAAGTC 3'
GADPH	Forward 5' ACTCCACGACATACTCAGCA 3'
	Reverse 5' CATCAACGACCCCTCATT3'

Supplementary Table 4: Comparison of renal function among HN rats and Febuxostat treated groups at different time points

	control	HN -1wk	HN-6wk	HN 1wk +Fx	Fx pretreatment
UA(umol/L)	56.00±6.70	104.33±19.63*	311.67±13.05**	104.00±10.2#	97.0±12.8#
sCr(μmol/L)	19.75±1.71	29.33±2.08**	50.00±8.73**	25.29±2.06#	24.58±1.72#
BUN(mmol/L)	5.78±0.34	8.65±0.78*	13.18±2.48**	6.36±0.69#	6.05±0.54#

*$p<0.05$, **$p<0.01$ for comparisons between the corresponding HN-6wk and control groups; # $p<0.05$ vs HN 1w+Fx rats or Fx pretreatment rats.
